# Magnetic Analysis of MgFe Hydrotalcites as Powder and Dispersed in Thin Films within a Keratin Matrix

**DOI:** 10.3390/nano13142029

**Published:** 2023-07-08

**Authors:** Franco Dinelli, Michele Modestino, Armando Galluzzi, Tamara Posati, Mirko Seri, Roberto Zamboni, Giovanna Sotgiu, Massimiliano Polichetti

**Affiliations:** 1 Istituto Nazionale di Ottica (INO) CNR, via Moruzzi 1, 56124 Pisa, Italy; 2 Dipartimento di Fisica, Università di Salerno, Via Giovanni Paolo II, 132, 84084 Fisciano, Italy; mmodestino@unisa.it (M.M.); agalluzzi@unisa.it (A.G.); 3 Istituto Superconduttori, Materiali Innovativi e Dispositivi (SPIN) CNR, Salerno, Via Giovanni Paolo II, 132, 84084 Fisciano, Italy; 4 Istituto per la Sintesi Organica e la Fotoreattività (ISOF) CNR, Via Gobetti 101, 40129 Bologna, Italy; tamara.posati@isof.cnr.it (T.P.); roberto.zamboni@isof.cnr.it (R.Z.); giovanna.sotgiu@isof.cnr.it (G.S.); 5 Istituto per lo Studio dei Materiali Nanostrutturati (ISMN) CNR, Via Gobetti 101, 40129 Bologna, Italy; mirko.seri@cnr.it; 6 Kerline srl, Via Gobetti 101, 40129 Bologna, Italy

**Keywords:** hydrotalcites, magnetic properties, NanoMOKE, PPMS-VSM, thin films, Keratin

## Abstract

Hydrotalcites (HTlcs) are a class of nanostructured layered materials that may be employed in a variety of applications, from green to bio technologies. In this paper, we report an investigation on HTlcs made of Mg and Fe, recently employed to improve the growth in vitro of osteoblasts within a keratin sponge. We carried out an analysis of powder materials and of HTlcs dispersed in keratin and spin-coated on a Si/SiO_2_ substrate at different temperatures. A magnetic study of the powders was carried out with a Quantum Design Physical Property Measurement System equipped with a Vibrating Sample Magnetometer. The data gathered prove that these HTlcs are fully paramagnetic, and keratin showed a very small magnetic response. Optical and Atomic Force Microscopy analyses of the thin films provide a detailed picture of clusters randomly dispersed in the films with various dimensions. The magnetic properties of these films were characterized using the Nano Magneto Optical Kerr Effect (NanoMOKE) down to 7.5 K. The data collected show that the local magnetic properties can be mapped with a micrometric resolution distinguishing HTlc regions from keratin ones. This approach opens new perspectives in the characterization of these composite materials.

## 1. Introduction

Hydrotalcites (HTlcs) are a class of nanostructured layered materials made of positively charged metal hydroxide layers compensated by exchangeable charge balancing anions [1,2]. They can be generally described with the following formula: [M(II)_1−x_ M(III)_x_ (OH)_2_]^x+^ [A_x/n_^n−^] mH_2_O. Here, M(II) is a divalent cation such as Mg, Ni, Zn, Cu, or Co; M(III) is a trivalent cation such as Al, Cr, Fe, or Ga; A^n−^ is an anion of charge n; and m is the molar amount of co-intercalated water. Due to the presence of large interlayer spaces and to the large number of exchangeable anions, HTlc may act both as a good ion exchanger and as a good absorbent. These characteristics clearly mark them as potentially useful in a variety of applications, ranging from green to bio technologies [3,4,5,6].

Some categories of HTlcs may thus be used in biological applications [7,8,9]. To do so, a high level of biocompatibility is needed, and the possible selection of metal atoms is strongly limited. However, thanks to the fact that the metal atoms forming the anionic layers M(II) and M(III) can vary in a wide range, a suitable choice is not difficult to find. Additionally, this choice at the production stage also allows the properties of the particles to be selected. For instance, one can obtain magnetic nanoparticles when the atoms include Ni and Fe and other couples.

Keratins are naturally abundant non-food proteins found, for instance, in hair, wool, horns, nails, and feathers. Wool keratin has been widely employed in tissue engineering and wound healing applications due to its biodegradability, biocompatibility, and bioactivity [10,10,11,12,13]. In a recent publication, keratin sponges containing magnetic HTlc nanoparticles were used to prepare innovative 3D scaffolds [14]. These HTlcs were synthesized by coupling Mg and Fe, which, besides a high biocompatibility, present magnetic properties. The scaffolds obtained were employed for experiments on osteoblasts under the effect of a static magnetic field externally applied. The results showed an enhancement of cellular activity. MgFe HTlcs have been already used in the past, namely for green applications [15,16]. Though their structure and composition have been well characterized [17], they have not been fully characterized in terms of their magnetic properties.

In the first instance, the single components need to be tested separately. Keratin and HTlc powders have been analyzed using a Physical Property Measurement System (PPMS) with a Vibrating Sample Magnetometer (VSM) option [18] that allows the measurement of magnetization versus a static field in a temperature range from 300 down to 2.5 K. Secondly, HTlcs need to be locally studied in a real environment, i.e., the keratin matrix used as scaffolds. To do this, they were dispersed in a keratin water solution, as for the sponge fabrication, and the solution was spin-coated on pieces of Si/SiO_2_ wafers. In this way, the magnetic properties of the samples could be locally analyzed with the help of a Magneto-Optic Kerr Effect (MOKE) technique [19].

These films have also been investigated with Optical Microscopy to visualize the distribution of HTlcs, and with Atomic Force Microscopy (AFM) to map the film topography. These data can be finally correlated with the reflectivity maps obtained with a DMO NanoMOKE^®^ and, with the Kerr rotation values, correlated with the local magnetic behavior. Our results indicate that MgFe HTlc powder is fully paramagnetic down to 2.5 K. Once dispersed in the keratin matrix and spin-coated in films, the HTlcs present themselves in clusters of various dimensions. They still have a paramagnetic behavior down to 7.5 K. Keratin is weakly paramagnetic, as determined with NanoMOKE, although diamagnetic regions can be found at the interfaces between HTlc clusters and keratin. These data are fully described in the following Section 3, and an interpretation of the results is provided in the Section 4.

## 2. Materials and Methods

MgFe-HTlc nanoparticles, with an average diameter of around 30 nm, were prepared using the double-microemulsion water-in-oil technique, as described in the literature [1]. They can be represented by the following formula: [Mg_0.76_Fe_0.24_(OH)_2_]Br_0.12_(CO_3_)_0.06_∙0.65H_2_O. Briefly, two microemulsions were prepared by dispersing 3.12 g of cetyltrimethylammonium bromide (CTAB), 3.9 mL of n-butanol, 9 mL of isooctane, and 3.4 mL of aqueous phase. The aqueous phase of the first microemulsion consisted of Mg (NO_3_)_2_ 0.4 M and Fe (NO_3_)_3_ 0.125 M. The aqueous phase of the second microemulsion was an NH_3_ solution of 5.0 M. Equal volumes of the two microemulsions were then mixed to obtain the precipitation of HTlc in the reverse micelles. The resulting system was aged at 70 °C for 16 h. Afterwards, the nanoparticles were recovered by centrifugation (12,000 rpm, 10 min) and the precipitate was washed with an ethanol–chloroform mixture (1:1 *v*/*v*) (3 × 30 mL) and then with water (3 × 30 mL).

High-molecular-weight keratin powder (≈50 kDa) extracted from raw wool was kindly donated by Kerline Srl (Bologna, Italy). The HTlc/keratin films were prepared by spin-coating (in air) a water solution on Si/SiO_2_ substrates previously plasma-treated for 3 min. The solution contained 10% of keratin and 2.5 or 5% of HTlc powder in weight percentages. The spinning conditions were as follows: 4000 rpm, acceleration 4000 rpm/s, 60 s. No thermal treatments were carried out. The final thickness was around 220 nm.

The Scanning Electron Microscope (SEM) data of the HTlc/keratin films were collected with a Zeiss EVO LS 10 LaB6 (Carl Zeiss, Milano, Italy).

The X-ray diffraction patterns (XRD) of the HTLc powder were obtained with a Philips X’Pert PRO MPD diffractometer (Philips, Milano, Italy), equipped with a Cu Kα radiation source (λ = 0.15406 nm), operating at 40 kV and 40 mA, and an X’Celerator detector. The diffractograms were registered in the 2θ configuration: with a range between 3 and 70°, a step size of 0.033°, and a scan time of 30 s per step. Data were elaborated with the HighScore software (version 5.1). 

The HTlc powder was characterized using the PPMS (from Quantum Design, Rome Italy) of a Vibrating Sample Magnetometer (VSM). The magnetic moment can be measured as a function of the temperature M(T), the magnetic field M(B), and the time M(t). To perform the M(T) measurement, the sample was cooled down to 2.5 K in the absence of a magnetic field. After this, a magnetic field of 100 mT was applied and the sample was warmed up to 300 K to obtain the Zero Field Cooling curve (ZFC). Subsequently, it was cooled again at 2.5 K to perform the Field Cooling curve (FC). For the M(B) measurements, the target temperature was fixed, and the magnetic field was swept from 0 T to 9 T, from 9 T to −9 T, and again from −9 T to 9 T. For more details about the sequences of the measurements, please refer to Reference [18].

NanoMOKE^®^ (Durham Magneto-Optics, Durham, UK) is an instrument based on the Magneto-Optic Kerr Effect (MOKE). It combines the capabilities of the Durham Magneto-Optics NanoMOKE3 with the flexibility of a closed-cycle optical Cryostation. This system allows characterization of the surface magnetic properties of the samples from room to low temperatures with a few micrometers of lateral resolution. The experimental setup was based on a scanning laser microscope operating at λ = 660 nm with an integrated optical system made of a pair of galvanometric mirrors. The measurements were performed in a longitudinal configuration, i.e., the external magnetic field was applied parallel to the sample surface (in-plane magnetization).

Atomic force microscopy (AFM) was performed using a hybrid system that was assembled using a commercial head (SMENA, NT-MDT, Moscow, Russia), home-built electronics, home-developed software, and a commercial digital lock-in amplifier (HF2LI, Zurich Instruments, Zurich, Switzerland) [20]. The setup was operated in intermittent contact mode (ICM). The cantilevers employed are commercially available from MikroMasch (Tallinn, Estonia) as the HQ:NSC35 model (nominal force constant from 5.4 to 16 N/m and resonance frequency from 130 to 300 kHz). 

## 3. Results

**Figure 1:**
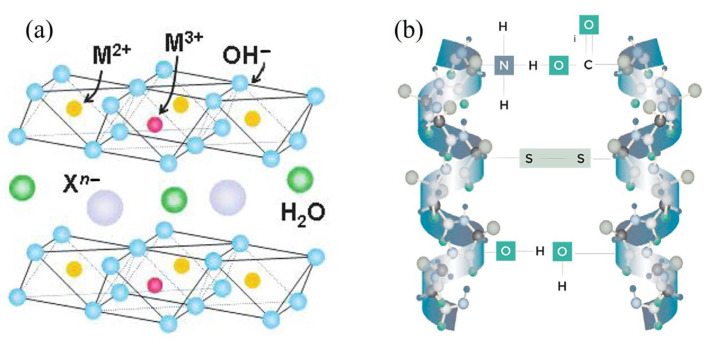
(**a**) Sketch of the structure of MgFe HTlc; (**b**) 3D representation of the keratin molecule.

In Figure 1, we report in (a) a sketch of the structure of the HTlcs, and in (b) the schematic representation of keratin. As already mentioned, the HTlcs synthesized for this research were made with Mg and Fe to create a magnetic biocompatible structure. The keratin employed was derived from wool and soluble in water.

**Figure 2:**
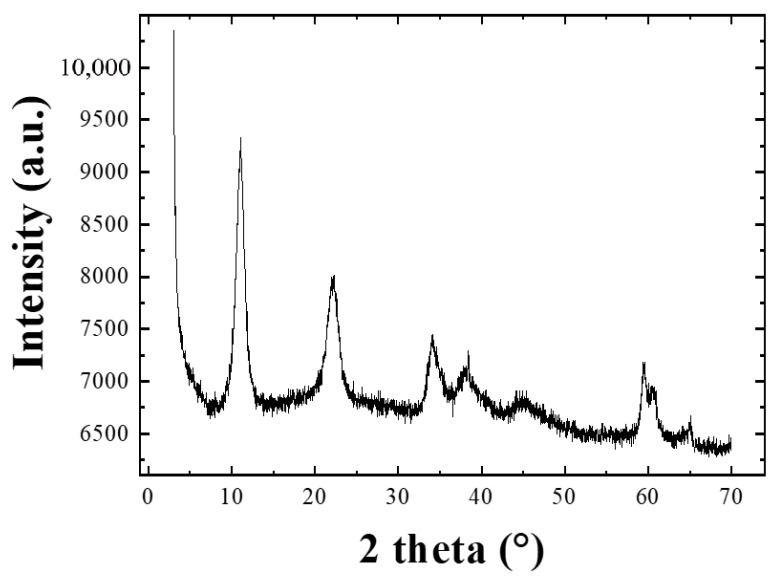
X-ray diffraction pattern of the HTLc powder.

In Figure 2 we show an XRD pattern of the synthesized powder. The 2θ plot indicates that this material has a lamellar structure with an interlayer distance of 8.06 Å, typical of the MgFe HTlc and compatible with the presence of bromide anions in the interlayer regions [17].

Then, we characterized the magnetic properties of the single materials in the powder form. In our case, this consisted of considering a portion of the MgFe HTlc powder and an equivalent portion of keratin powder. These portions of materials were separately inserted into the sample holder of a PPMS-VSM and measured. 

**Figure 3:**
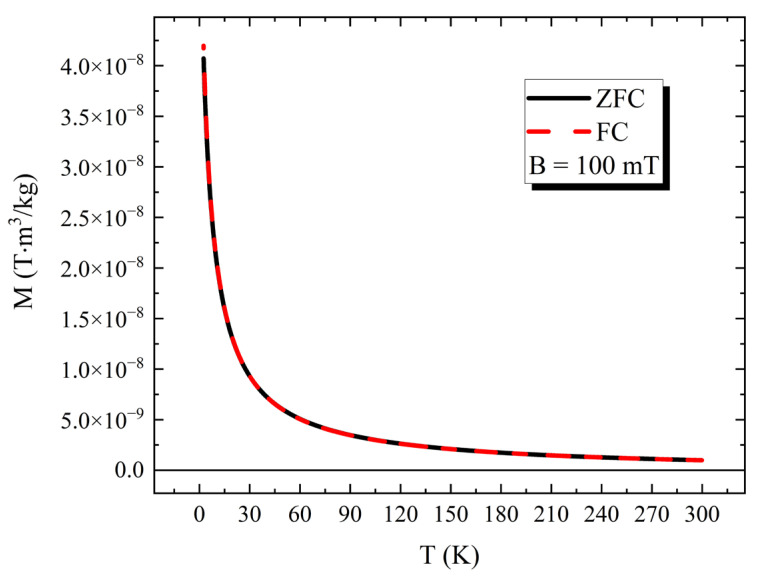
Magnetization (M) versus Temperature (T) plot of PPMS-VSM data obtained on HTlc powder, applying a static field of 100 mT, from T = 2.5 to 300 K.

In Figure 3, we present a graph of the magnetization (M) versus temperature (T) obtained with the PPMS using the VSM option. The data show that the behavior of the HTlc powder appears to be paramagnetic, with M continuously increasing as T reduces to 2.5 K, and with the Zero Field Cooling (ZFC) and the Field Cooling (FC) curves fully overlapping. 

**Figure 4:**
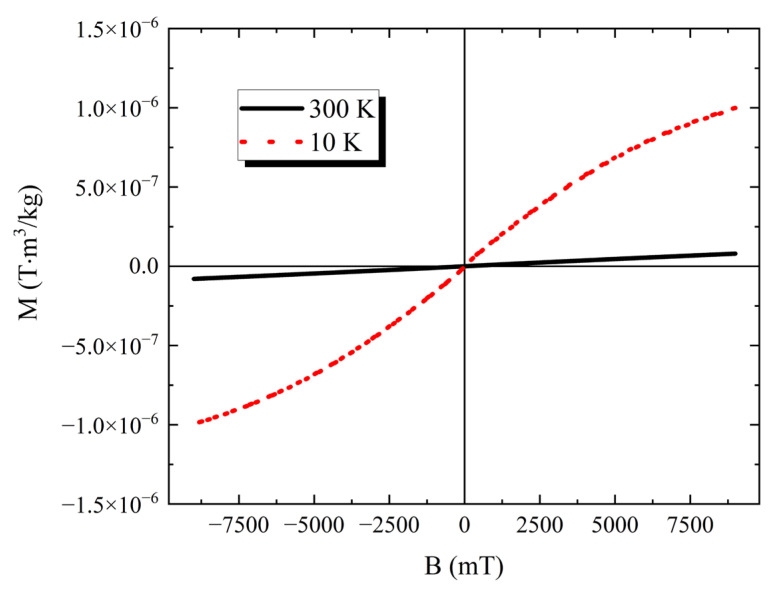
Magnetization (M) versus Field (B) plots of PPMS-VSM data obtained on HTlc powder, applying a static field from −9000 to 9000 mT, at T = 10 and 300 K.

In Figure 4, we present the plots of M versus the magnetic field (B) obtained at 10 and 300 K. At 300 K, the curve is linear with a smaller signal amplitude, whereas at 10 K the amplitude is much larger, and the curve slightly bends at high B values. However, the cycle does not present hysteresis, coherent with paramagnetic behavior. This is valid down to T = 2.5 K.

The same experiment carried out on keratin powder did not provide a clear indication of its magnetic behavior. Keratin showed a very weak signal and the magnetic contribution from the plastic sample holder was comparable with, and thus covers, it at all T values.

After that, we characterized the properties of spin-coated thin films obtained from water solutions of HTlc and keratin. The film thickness was around 220 nm for all the samples.

**Figure 5:**
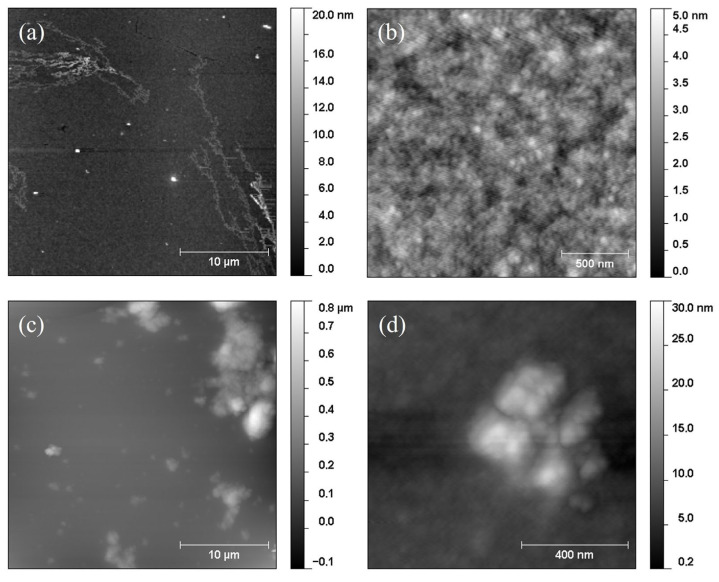
AFM images showing the morphology of (**a**,**b**) a keratin film and (**c**,**d**) a 2.5% 220 nm sample.

In Figure 5, we present some topographical images acquired with Atomic Force Microscopy (AFM) of: (a,b) a keratin film and (c,d) keratin mixed with 2.5% of HTlc powder in weight. Pure keratin has a very flat surface that is only characterized by the presence of some dendritic motives. These features have a thickness of a few nanometers, probably due to local reorganization of the molecules at the free surface determining some crystallization.

All the HTlc/keratin films instead had a much higher roughness for both a HTlc concentration of 2.5 and 5% in weight. Focusing on the morphology of the 2.5% sample, we observed several clusters whose height ranged from a few tens up to hundreds of nanometers. These clusters have various dimensions and emerge from the surrounding keratin matrix. Though the preparation methods are different, the SEM images reported in Ref [14] indicate that the distribution and cluster dispersion may be similar to the case of HTlcs dispersed in sponge scaffolds used for osteoblast growth. 

**Figure 6:**
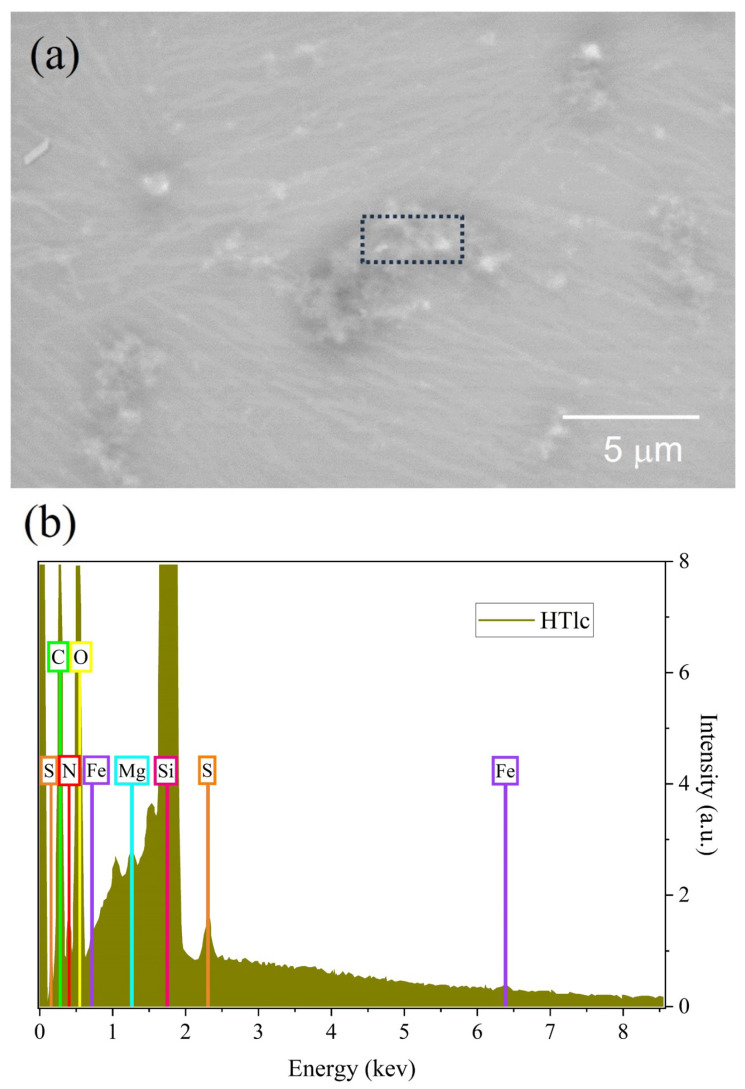
(**a**) SEM image of a 2.5% 220 nm sample. (**b**) EDX spectrum carried out in the area defined by the black dotted rectangle.

In Figure 6, we report measurements performed on the same sample with an environmental SEM. In image (a), the presence of the dendritic motives observed with AFM, and attributed to keratin, can still be observed. HTLc clusters of various dimensions are also visible. In particular, it is relevant to underline that these clusters show various morphologies, such as card-of-house and sand-rose structures. These structures may show different internal organization and relative distance of the single HTLc.

An energy-dispersive X-ray (EDX) analysis was also performed in the region defined by the black dotted rectangle, where a large cluster could be localized. The EDX spectrum shows several peaks, besides the predominant one due to the Si substrate. Some may be reconducted to Mg and Fe atoms on the one side, while others to C, O, N and S atoms that may be attributed to keratin. This indicates that these structures are due to clustering of HTLcs as expected, and that they are mixed with keratin.

**Figure 7:**
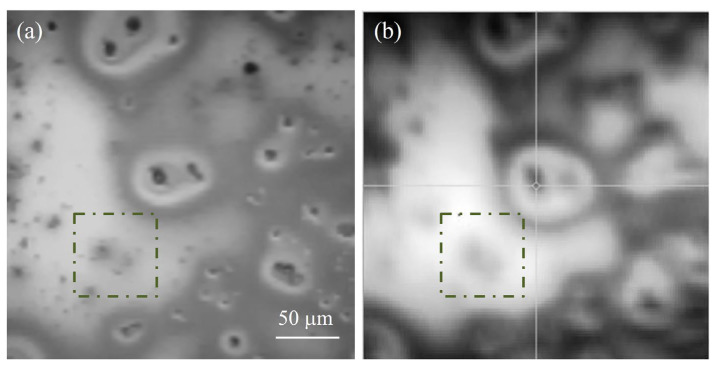
A comparison between (**a**) an optical image and (**b**) a NanoMOKE reflection map of the same area on a 2.5% 220 nm sample.

In Figure 7, we report two pictures of the same area of a 2.5% sample. They are (a) an optical microscopy image and (b) a reflectivity map, recorded with NanoMOKE, that was obtained by scanning the laser spot over the surface while recording the reflectivity. This map shows large variations in reflectivity from area to area because the clusters are dark. The optical image helps to correlate the reflectivity data with the presence of clusters. One further consideration needs to be immediately made: the reflectivity data of the films with clusters, obtained with NanoMOKE, are not as accurate as the optical data. This is due to the laser spot that has a finite lateral dimension, as it is the result of focalization. Its size is indicated by the manufacturer to be a diameter of a few microns. From this comparison, we can divide the reflectivity maps into three regions, depending on the reflectivity value: low values—larger HTlc clusters; intermediate values—mixed areas, i.e., keratin with smaller clusters; and high values—keratin only, possibly with some scattered nanoparticles not detectable either with optical microscopy or AFM.

We now present the data obtained from an analysis of the various films with NanoMOKE. Our instrument can operate from 300 K down to 7.5 K. MOKE is the only instrument available that can map the local magnetic properties of a surface and therefore is suitable for filmed materials with good reflectivity [19]. The software allows one to measure the Kerr rotation due to the application of an external magnetic field, that, in the configuration we have used, is parallel to the plane of the sample surface. 

**Figure 8:**
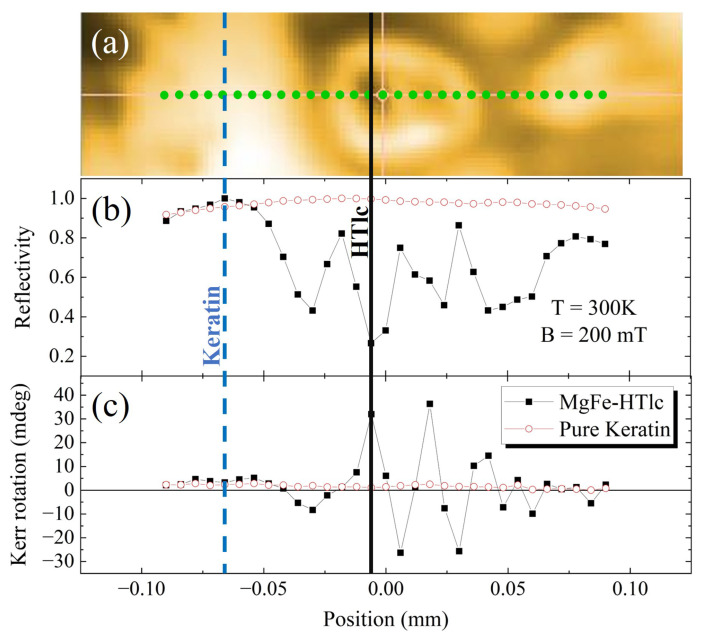
NanoMOKE data recorded scanning along a single line (in green) at 300 K. (**a**) Reflectivity map. Profiles of (**b**) reflectivity and (**c**) maximum Kerr rotation versus position of a keratin film (red line) and a 2.5% 220 nm sample (black line).

In Figure 8, we first report a line scan performed at 300 K on the region (a) already shown in Figure 7. The acquisition was performed with a pixel distance of 4 microns. This region was selected to include clusters of different dimensions and some keratin-only areas (black line). In the graphs, we also report data from a line of the same length and pixel distance on a film of keratin (red line). Thus, we have plotted (b) the reflectivity and (c) the maximum Kerr rotation measured at the maximum field applied (200 mT) for a keratin film and a 2.5% 220 nm sample.

The maximum Kerr rotation was obtained on large clusters. They have a peak value of around 20 mdeg, whereas the keratin-only region on the left shows a much smaller signal. These data agree with what is observed on powders. They also prove not only that NanoMOKE can identify clusters and keratin, but that it can provide a wealth of information. In particular, the instrument is sensitive to the local properties that may change at the interfaces of clusters and keratin. 

**Figure 9:**
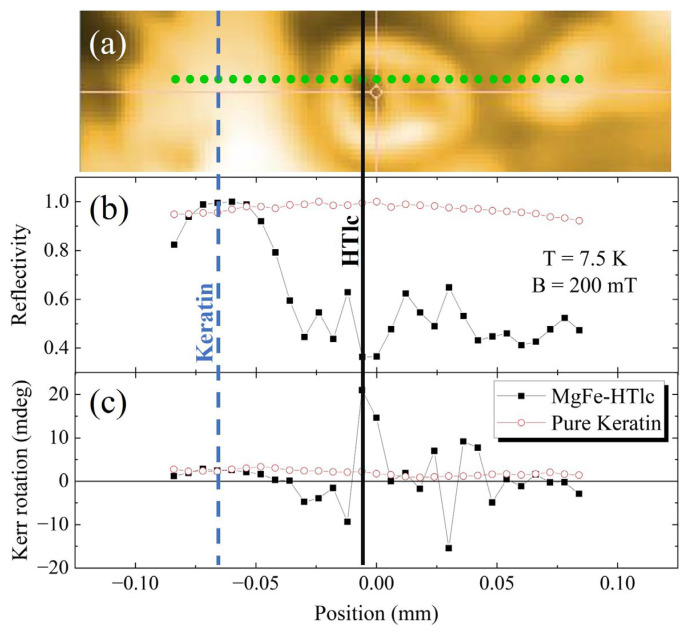
NanoMOKE data recorded scanning along a single line (in green) at 7.5 K. (**a**) Reflectivity map. Profiles of (**b**) reflectivity and (**c**) maximum Kerr rotation versus position of a keratin film (red line) and a 2.5% 220 nm sample (black line).

In Figure 9, we then report data obtained from a line scan carried out in the same regions as in Figure 8 but at 7.5 K. The trend of the lines can be considered substantially similar both for the clusters and for the keratin regions. The little discrepancies measured in reflectivity and Kerr rotation may be due to the uncertainty in choosing and stabilizing the sampling position. 

**Figure 10:**
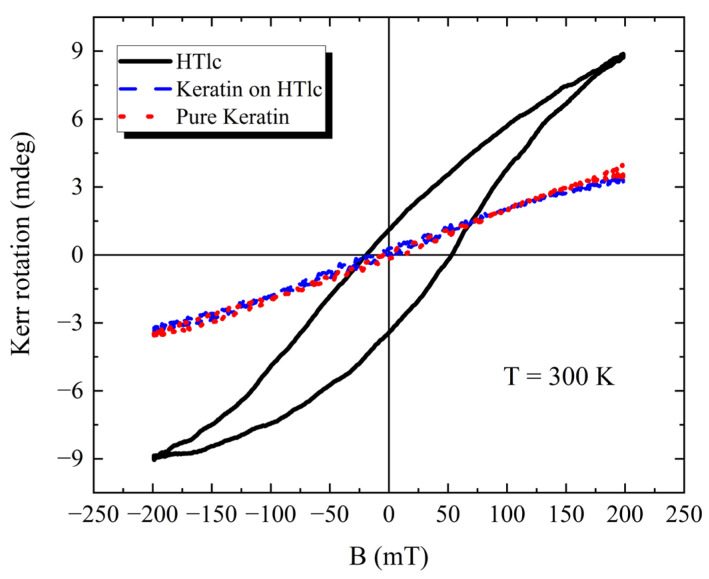
Kerr rotation versus B plots of three regions at 300 K: a pure keratin film, keratin only in a 2.5% 220 nm sample, and clusters in a 2.5% 220 nm sample.

In Figure 10, we show the characteristics of the entire cycle, i.e., Kerr rotation versus B curve for three specific regions at 300 K: a pure keratin film, keratin only in a 2.5% 220 nm film, and clusters in a 2.5% 220 nm film. The data obtained for the keratin film show a low signal, very similar to the keratin-only regions. The regions with larger clusters present a much higher signal, which is in full agreement with the powder data with PPMS-VSM.

**Figure 11:**
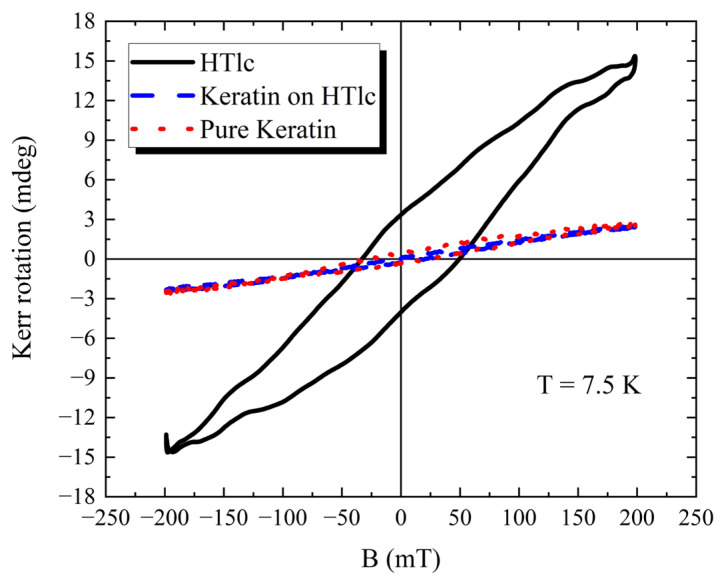
Kerr rotation versus B plots of three regions at 7.5 K: a pure keratin film, keratin only in a 2.5% 220 nm sample, and clusters in a 2.5% 220 nm sample.

In Figure 11, we present the Kerr rotation versus B curve for the same three regions reported in Figure 10 but recorded at 7.5 K. These data are very similar to the ones collected at 300 K. The only major difference is represented by an increase of the maximum Kerr rotation for the HTlc cluster. The little discrepancies measured in the Kerr rotation may be due to the uncertainty of the sampling position.

**Figure 12:**
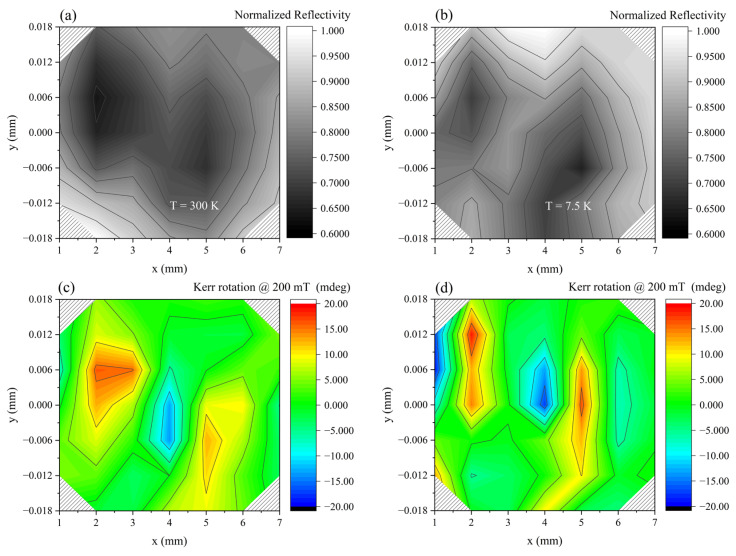
NanoMOKE data recorded on a square region of a 2.5% 220 nm sample at 7.5 and 300 K; see Figure 7. (**a**,**b**) Reflectivity maps. (**c**,**d**) Maps in false colors of the maximum Kerr rotation at B = 200 mT.

In Figure 12, we finally present two bidimensional maps obtained on the area indicated with a square in Figure 7. In (a,b) we report the reflectivity maps of the same region where the acquisitions were made. The measurements were performed at (c) 300 K and (d) 7.5 K. This area was chosen as we wanted to focus our investigation on relatively small clusters that are embedded in the matrix, avoiding the large ones, which may have a more complex behavior due to the shape of the cluster.

These maps were plotted using a false color representation that shows the maximum Kerr rotation measured at given x and y location for a field of 200 mT. The color bar visible on the right-hand side indicates the correspondence between color and maximum Kerr rotation. Sampling was performed with a pixel distance of 6 microns. The maps are quite clear in evidencing a stronger Kerr rotation of the clusters and a weaker one of the keratin-only regions. 

The other parts shown in the map have a more complex contrast: the signal value sometimes corresponds to a negative Kerr rotation that could be associated with a diamagnetic behavior, like in the case of the area located in the middle of the clusters. This proves once more the fact that NanoMOKE is rather sensitive to variations from point to point and can provide detailed information of the local magnetic properties.

## 4. Discussion

As already underlined, for a complete study of nanoparticles, such as HTlcs, to be employed within a polymer matrix in a composite structure, one needs to start from a careful analysis of the single components. In our case, the powder behavior of the MgFe HTlcs can be clearly described, as the powder shows a fully paramagnetic trend that is maintained from 300 down to 2.5 K. In addition, the M versus B curve does not show hysteresis at both 300 and 10 K. These are interesting observations in themselves. Other magnetic HTlc, like those made with Ni and Fe, show a super-paramagnetic behavior, i.e., the M versus T plot presents a discontinuity at a few K, which can be identified with a blocking temperature T_B_ [21]. This result might be an indication that the overall properties of the MgFe HTlc are determined by the magnetic moment of the single atoms.

On the other hand, keratin powder has a very weak response that only with NanoMOKE can be identified as paramagnetic. It is relevant to consider that the transmittance data of keratin in foils indicate that it is partially transparent [22], around 70% in the red region of the wavelength. This is even more important when keratin is employed as a thin film. We can then assume that our thin films allow the transmission of a laser through the whole thickness, thus sampling both keratin and the Si/SiO_2_ substrate. However, we have found that Si/SiO_2_ substrates have a weak diamagnetic signal, as is reported in the literature for smooth Si/SiO_2_ interfaces [23]. This is evidence that the keratin response is dominating the whole signal at all T values. In the literature available on this topic, we have only been able to find a paper that reports measurements performed with EPR and that indicates that a keratin molecule has paramagnetic centers [24].

In the HTlc/keratin films, the locations where keratin or the HTlc clusters are prevalent, the Kerr rotation versus B curves are in full agreement with the data obtained for the relative powders. Thus, a complete correlation between the PPMS-VSM graphs and the NanoMOKE maps has been found, both for the keratin and cluster regions. The main difference is, however, represented by the hysteresis of the Kerr rotation versus B curves. The PPMS-VSM curves do not show any opening of the cycle, whereas the NanoMOKE curves do at 300 K and 7.5 K. To explain this observation, it is relevant to clarify that the two techniques operate into two different time regimes. Specifically, the rate of the amplitude variation of the external field applied is quite different for the two cases: 1600 mT/s for the NanoMOKE and only 10 mT/s for PPMS-VSM. Therefore, they differ by more than two orders of magnitude. This might explain the differences in the curves, i.e., the system might not be able to fully relax magnetically when NanoMOKE experiments are performed.

Finally, the complexity of the data that can be contained in a NanoMOKE map provides enough evidence for the high sensitivity of this technique. The reflectivity map shown in Figure 12 presents a bright location in between two dark ones (where some HTlc clusters are located) where the Kerr rotation becomes strongly negative. A negative value of the Kerr rotation can be generally associated with diamagnetism. Nevertheless, the materials forming the films are not diamagnetic when tested separately. A possible explanation might be that in this area keratin thins down and we are sampling the Si/SiO_2_ substrate. The data we have obtained on the substrate are, however, much lower in absolute value. Therefore, we suggest that the magnetic field generated by clusters with different morphologies, that is, different internal organization and relative distance of the single HTLc, may combine to produce such a value of Kerr rotation. Though this complexity cannot currently be fully accounted for, one can argue that this technique is rather powerful as it indicates local phenomena not easily predictable in advance. Further theoretical and experimental work is, however, necessary to fully explain the physical phenomena that determine the whole variety of the information.

## 5. Conclusions

In this study, we have employed a range of different experimental techniques to characterize the magnetic properties of powder MgFe HTlcs and thin films of MgFe HTlcs dispersed in a keratin matrix. Specifically, our approach combines the use of a PPMS using the VSM option for the magnetic analysis of powder down to 2.5 K, and NanoMOKE to magnetically map the surface of thin films with a few micrometers’ resolution down to 7.5 K. In addition, we have also employed Optical and Atomic Force Microscopy to investigate the morphological properties of the studied film surfaces and correlate them with the magnetic ones.

Our findings show that keratin, a biocompatible material, presents a very weak paramagnetic behavior in films at all temperature values. On the other hand, MgFe HTlcs synthesized for bio applications have a fully paramagnetic behavior in powder, but also when dispersed in a keratin matrix. In particular, NanoMOKE allows mapping thin films with a remarkable lateral micrometric resolution and a high sensitivity, providing a wealth of details with regard to the local magnetic behavior. This type of composite is increasingly used, for instance, in bioengineering as herein reported, but also in other applications. Thus, our approach represents a powerful one, viable for future investigations of other HTlc and magnetic nanoparticles, isolated or mixed within polymeric matrixes.

## Data Availability

Data will be provided upon request to the correspondent authors.
